# Evidence for high-density liquid water between 0.1 and 0.3 GPa near 150 K

**DOI:** 10.1073/pnas.1819832116

**Published:** 2019-03-28

**Authors:** Josef N. Stern, Markus Seidl-Nigsch, Thomas Loerting

**Affiliations:** ^a^Institute of Physical Chemistry, University of Innsbruck, A-6020 Innsbruck, Austria

**Keywords:** polyamorphism, high-density amorphous ice, very high-density amorphous ice, glass-to-liquid transition, high-density liquid water

## Abstract

Thermal stability against crystallization upon isobaric heating at pressure 0.1 ≤ *P* ≤ 1.9 GPa is compared for five variants of high- (HDA) and very high-density amorphous ice (VHDA) with different preparation history. At 0.1–0.3 GPa expanded HDA (eHDA) and VHDA reach the same state before crystallization, which we infer to be the contested high-density liquid (HDL). Thus, 0.3 GPa sets the high-pressure limit for the possibility to observe HDL for timescales of minutes, hours, and longer. At *P* > 0.3 GPa the annealed amorphous ices no longer reach the same state before crystallization. Further examination of the results demonstrates that crystallization times are significantly affected both by the density of the amorphous matrix at the crystallization temperature *T*_*x*_ as well as by nanocrystalline domains remaining in unannealed HDA (uHDA) as a consequence of incomplete pressure-induced amorphization.

## Significance

The two-liquid model is appealing to explain the anomalous nature of supercooled water. However, it is still contested whether or not the amorphous polymorphism at cryoconditions develops into a liquid–liquid transition at higher temperatures. While some work has been devoted to this question at ambient pressure conditions, there are barely any experiments at high-pressure conditions. Here we reveal a temperature window, in which bulk high-density liquid water (HDL) can be accessed prior to crystallization at 0.1–0.3 GPa based on path independence. By contrast, at higher pressures crystallization takes place from the amorphous solid state. This paves the way for future exploration of the liquid–liquid transition from HDL to low-density liquid water at thermodynamic equilibrium near 0.1 GPa.

## Polyamorphism and Its Implications

High-density amorphous ice (HDA) was discovered in 1984 (1) and suggested to be a second form of amorphous ice, distinct from the previously known low-density amorphous ice (LDA) (2, 3). The existence of more than one solid disordered state for water and the possibility to reversibly switch back and forth between the two (4) prompted vivid discussions about the physical implications. Prominently, it raised the question if there might also be two distinct liquid phases thermodynamically connected to the amorphous forms. In fact, several scenarios involving two distinct liquids have been proposed to explain water’s anomalous nature. The reason why it has thus far been difficult to resolve this issue is due to the fact that the region of interest is situated in an area in the phase diagram in which disordered states are not stable, the so-called “no man’s land” of water. No matter if coming from the high-temperature supercooled liquid’s or the low-temperature amorphous ices’ side, the timescales of crystallization become so fast that an equilibration of liquids is impossible in the experimental time frame (5).

One essential aspect in this context is the question if the amorphous ices can actually be judged as distinct disordered states. It has been demonstrated that the state originally obtained by Mishima et al. (1) by pressure-induced amorphization (PIA) at low temperatures is not a homogeneous and well-characterized amorphous state (6), but that it contains (nano)crystalline, distorted hexagonal domains remaining from the initial phase of hexagonal ice (7, 8). When this high-density state (since labeled unannealed HDA, uHDA) is heated under pressure (*P* ≤ ∼0.5 GPa) it relaxes to a slightly less dense, presumably more structurally homogeneous state (expanded HDA, eHDA) (9). A third amorphous state of even higher density was reported to form when HDA is heated at *P* ≥ ∼0.8 GPa to a temperature just below the crystallization, namely very high-density amorphous ice (VHDA). Similar to the situation regarding LDA and HDA, a controversial discussion whether the transition between HDA and VHDA has to be considered continuous or discontinuous or if they should be judged two distinct polyamorphs is still ongoing (5, 10).

## Glass-To-Liquid Transition of HDA

Attempts to learn more about the nature of supercooled water through cooling of the liquid are hampered by the high-temperature boundary of no man’s land, the homogeneous nucleation temperature. However, it has been demonstrated that the deeply supercooled liquid state may also be assumed before crystallization when heating the amorphous ices. At ambient pressure this has in fact been shown for LDA (11, 12) and eHDA (13) where calorimetric glass-to-liquid transitions were identified. Water’s second glass transition pertaining to high-density liquid water (HDL) at 1 bar has opened an experimental window between ∼113 and 135 K in which HDL can be studied experimentally. At elevated pressures various experiments were conducted to obtain glass transition temperatures *T*_*g*_. Mishima measured endothermic events upon decompression of emulsified HDA and attributed the findings to a glass-to-liquid transition onset (14). The isobaric thermal expansivity of eHDA was investigated in volumetric experiments by Seidl et al. (15). When heating eHDA at pressures 0.1–0.3 GPa isobarically they were able to observe a kink in the volume-versus-temperature curve before crystallization which proved to be reversible upon cooling and reheating, allowing them to define volumetric values of *T*_*g*_. Handle et al. (16) probed the state of relaxation in HDA samples as a function of annealing temperature and time at 0.1 and 0.2 GPa. From shifts of the transition exotherms to LDA caused by annealing at high pressure they derived *T*_*g*_ values ∼10 K above those obtained by Seidl et al. (15). However, Handle et al. state that the volumetric values for *T*_*g*_ by Seidl et al. are within their own results’ experimental error margin. In a more recent study on volume relaxation processes, Handle and Loerting (17) obtained *T*_*g*_ values for HDA and VHDA in a much broader pressure range of 0.1–1.6 GPa by applying a fit function to isobaric heating curves. They were able to disentangle contributions to the volume changes originating from relaxation and crystallization––and hence to define a volumetric *T*_*g*_. The results are in good agreement with the previously reported values of *T*_*g*_. That is, in the pressure range 0.1–0.4 GPa water’s second glass transition is located between 130 and 160 K, in the vicinity of the crystallization temperature *T*_*x*_. At 1.0 GPa, Andersson and Inaba report dielectric relaxation times of 100 s at 122 K, but conclude that the actual *T*_*g*_ should be at higher temperatures (18). At the same pressure Andersson reports *T*_*g*_ from *c*_p_ measurements to be around 140 K (19). In this pressure region (*P* ≥ 1.0 GPa), the results by Handle and Loerting (17) agree well with these studies.

## Aim of This Study

Our work aims at comparing crystallization temperatures *T*_*x*_ and crystallization times *t_cryst_* for several variants of HDA and VHDA, differing in terms of their preparation history. Of particular interest is the state assumed just before crystallization––we want to understand whether this state is the contested HDL, or not. HDL can only be attained if the timescales for equilibration are shorter than for crystallization (5, 20, 21). For this reason crystallization times need to be maximized by elimination of additional channels of crystallization, which are known to be present in uHDA (8, 20). In our previous works on this topic (22, 23) we demonstrated that by varying the preparation route for VHDA (namely at a higher pressure of 1.9 GPa and a higher temperature of 175 K) it is possible to produce an amorphous state largely free of crystalline remnants (23). We demonstrated that this preparation protocol results in the thermally most stable amorphous ice at *P* ≥ 0.7 GPa. In this work we examine the crystallization behavior of several variants of uHDA, eHDA, and VHDA in a comparable and conclusive manner. Unlike in our previous studies on the topic (22, 23) we here choose a heating rate of 2 K min^−1^ to make our results directly comparable with the data by Seidl et al. (8, 20) and Salzmann et al. (24). Furthermore, the pressure range is extended both to lower (*P* < 0.7 GPa) and higher pressures (*P* > 1.8 GPa) (22, 23). We want to clarify under which conditions HDL can be prepared by heating amorphous ices under pressure close to *T*_*x*_.

## Results and Discussion

### Crystallization Onset Temperatures *T*_*x*_.

**Fig. 1.**
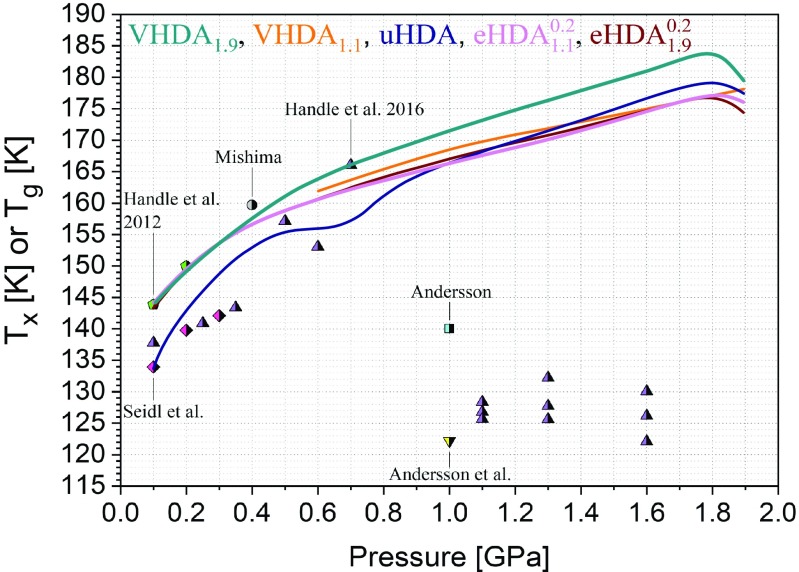
Summary of the crystallization temperatures *T*_*x*_ evaluated by the procedure shown in *SI Appendix*, Fig. S4*A* (heating rate of 2 K min^−1^). The curves include literature data in cases of $\text{eHD}{\text{A}}_{1.1}^{0.2}$ and uHDA (8, 20). Symbols represent glass transition temperatures *T_g_* for HDA reported in literature: diamonds by Seidl et al. (15), pentagons by Handle et al. (16), the circle by Mishima (14), upward triangles by Handle and Loerting (17), the square by Andersson (19), and the temperature at which the dielectric relaxation time is on the order of 100 s at 1.0 GPa: the downward triangle by Andersson and Inaba (18).

*T*_*x*_(*P*) was determined for five differently prepared amorphous ices at 0.1–1.9 GPa by isobaric heating with a rate of 2 K min^−1^ (detailed preparation protocols of the amorphous ices in *Material and Methods*; labeling of the amorphous ices serves the purpose of marking the route of preparation). *T*_*x*_ shown in Fig. 1 generally increases with pressure for the amorphous ices up to 1.8 GPa, but decreases to 1.9 GPa (VHDA_1.1_ being the exception). This observation indicates the approach of the high-pressure limit up to which amorphous ices may exist metastably. For comparison, (V)HDA crystallizes upon isothermal compression to ice VII at 85 K and ∼2.5 GPa (25).

In Fig. 1 especially two lines meet the eye: *T*_*x*_(*P*) for uHDA at *P* < ∼0.9 GPa and *T*_*x*_(*P*) for VHDA_1.9_ at *P* > ∼0.3 GPa. The former line *T*_*x*_(*P* < ∼0.9 GPa), because it is considerably below all other lines and displays a bump. The lowered thermal stability of uHDA has already been noted in the work of Seidl et al. (8, 20) for pressures up to 0.5 GPa. They explain it by a significant amount of nanocrystalline ice embedded in the amorphous matrix, small enough not to be detectable by X-ray or neutron diffraction (8, 20). Even though crystalline remnants cannot be detected directly, indirectly they leave an imprint on *T*_*x*_ as the crystalline “seeds” do not have to nucleate but only need to grow as the temperature is increased. Compared with both nucleation and crystal growth in case of the relaxed amorphous states, this results in a lower kinetic barrier against crystallization for uHDA and thus, a lowered thermal stability. This seems to be the case up to 0.9 GPa as discussed above and was reported in our previous work for VHDA comparing uHDA with VHDA_1.1_ (22). The existence of structural inhomogeneity in the form of crystalline remnants buried within the uHDA matrix has recently also been suggested in a study of Shephard et al. (26). They described uHDA obtained from PIA of ice I at low temperatures as a derailed state along the polymorphic transition path from crystalline ice I to crystalline ice IV. Similarly, Martelli et al. (27) demonstrate in MD simulations that, even though HDA is indeed amorphous (lacking “polydispersed icelike structures”), there exist some small domains structurally reminiscent of ice IV. The bump in the uHDA line is reproducible and implies that near ∼0.5 GPa *T*_*x*_(uHDA) appears to approach *T*_*x*_(eHDA). However, at 0.5–0.7 GPa the lines of *T*_*x*_ separate again, only to reapproach at *P* > ∼0.7 GPa and finally merge at ∼0.9 GPa. We rationalize this bump in terms of transformations in the nanocrystalline seeds embedded in the matrix. Seidl et al. (20) as well as Tonauer et al. (28) have presented evidence for the formation of ice IX nuclei rather than distorted ice I_h_ nuclei near 0.4 GPa. Depending on the ice phases crystallizing these nuclei are more or less effective in enhancing crystal growth rates, so that the bump shape appears close to pressures where the embedded nanodomains experience a transformation and the composition of the crystallizing ice polymorphs changes. At *P* ≥ 0.9 GPa uHDA and eHDA seem to reach a similar state before crystallization based on their similar *T*_*x*_ values which, however, are about 5 K lower than the ones of VHDA_1.9_ (Fig. 1).

**Fig. 2.**
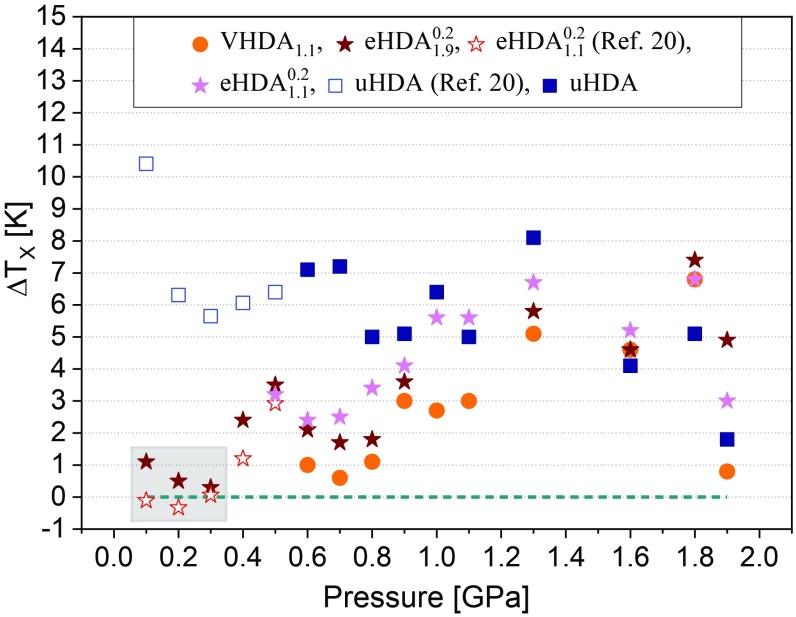
Difference in crystallization temperature Δ*T*_*x*_ of the various amorphous ices compared with *T*_*x*_(VHDA_1.9_), which defines the low-temperature border to no man’s land (dashed line).

The latter line *T*_*x*_(*P* < ∼0.9 GPa) meeting the eye relates to VHDA_1.9_ as it is considerably above all others at the high-pressure end. No other amorphous ice crystallizes later than VHDA_1.9_ at *P* > 0.3 GPa, and so we define this *T*_*x*_ line as the reference line for the highest thermal stability against crystallization that is experimentally accessible, i.e., as the low-temperature border to the no man’s land. The difference to this reference line is plotted in Fig. 2 as Δ*T*_*x*_. For uHDA it amounts to more than 10 K at 0.1 GPa and to 2 K at 1.9 GPa.

### Metastable Equilibrium for eHDA and VHDA at *P* ≤ 0.3 GPa and T < *T*_*x*_.

**Fig. 3.**
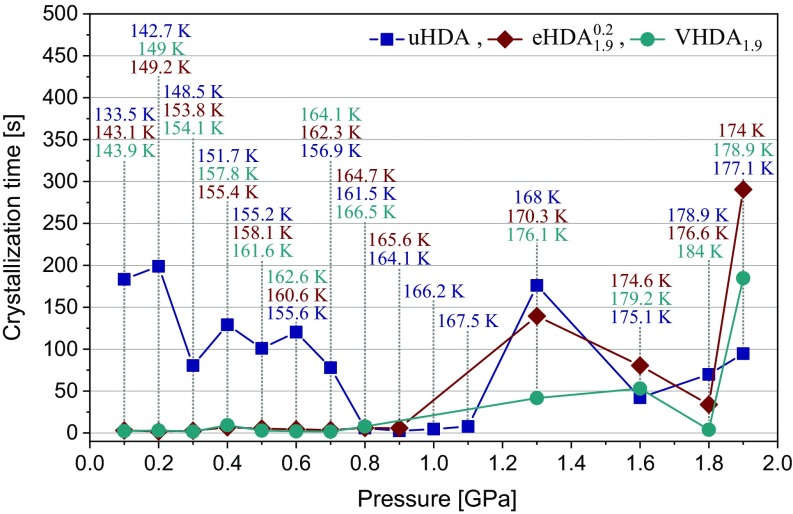
Crystallization times of uHDA, $\text{eHD}{\text{A}}_{1.9}^{0.2}$, and VHDA_1.9_, determined as described in the *Materials and Methods* (see also *SI Appendix*, Fig. S5). The temperatures correspond to *T*_*x*_ values of the amorphous ices at the respective pressures and are color coded accordingly.

The VHDA_1.9_ line is not only striking at the high-pressure end, but even more so at the low-pressure end. Intriguingly, it approaches all other *T*_*x*_ lines (except that of uHDA) and coincides at *P* ≤ 0.3 GPa. The precision of the match between these lines at pressures below 0.3 GPa is remarkable and is about ±1 K; see the framed region in Fig. 2. This is very close to the reproducibility of the method. In other words, there is a bifurcation point at ∼0.3 GPa below which the relaxed amorphous ices eHDA and VHDA crystallize at the same *T*_*x*_ independent from their previous experimental history, but above which they differ in *T*_*x*_. This implies the notion that at *P* ≤ 0.3 GPa an identical state is reached at T < *T*_*x*_ for all amorphous ices studied here (except uHDA). The sample preparation history no longer matters, even though we deal with inherently metastable amorphous ices. This change in phenomenology is explained by a nonequilibrium state crystallizing above 0.3 GPa, but an equilibrium state crystallizing below. The crystallization time of HDA itself does not change abruptly near 0.3 GPa (see Fig. 3 here and figure 4 in ref. 17).

In situ determinations of the glass transition temperature *T*_*g*_ at *P* ≤ 0.3 GPa (15–17) furthermore indicate the location of HDA’s *T*_*g*_ line to be close to or below the *T*_*x*_ lines of eHDA and VHDA presented in this work; see diamonds, pentagons, and upward triangles in Fig. 1. That is, the relation *T*_*g*_ < *T*_*x*_ appears valid at *P* ≤ 0.3 GPa. Since the identical state just before *T*_*x*_ is equilibrated in terms of volume and observed above *T*_*g*_ this strongly suggests the following explanation for these findings: volume relaxation in the sense of an *α*-relaxation is associated with *T*_*g*_, i.e., *T*_*g*_ defines a glass-to-liquid transition not a glass-to-glass transition. In other words, the identical state reached before *T*_*x*_ at *P* ≤ 0.3 GPa for four different sample preparations corresponds to the deeply supercooled liquid-state HDL. Obtaining the HDL state upon heating is not possible with uHDA as the embedded (nano)crystalline domains preclude structural equilibration at T < *T*_*x*_. These seeds render crystallization times shorter than equilibration times, thereby preventing access to the equilibrated liquid.

At *P* > 0.3 GPa the separation of the *T*_*x*_ lines of VHDA_1.9_ and eHDA (Fig. 1) suggests that timescales for crystallization become shorter than timescales for relaxation to the liquid (*T*_*g*_ > *T*_*x*_). That is, the amorphous ices crystallize before they can reach the deeply supercooled liquid state. This assumption is underlined by the *T*_*g*_ value deduced by Mishima at 0.4 GPa (14). It suggests that around 0.4 GPa *T*_*x*_ ∼ *T*_*g*_, and hence crystallization times and equilibration times are similar. At *P* ≥ 1 GPa the *T*_*g*_ values obtained by Andersson from *c*_p_ measurements (19) and by Handle and Loerting (17) from an analysis of volume relaxation are consistently about 30 or 50 K lower than *T*_*x*_(VHDA_1.9_). In the light of our observation that Δ*T*_*x*_ ∼ 6 K between VHDA_1.9_ and eHDA (see star at 1.0 GPa in Fig. 2) we suggest that the *T*_*g*_s determined at *P* ≥ 1 GPa (17, 19) are not due to a glass-to-liquid, but rather due to an orientational glass transition, by contrast to the *T*_*g*_s at 0.1–0.4 GPa. This is because equilibrated HDL has a defined *T*_*x*_, no matter which amorphous ice it has originated from, i.e., the condition Δ*T*_*x*_ = 0 K needs to hold for HDL. These conclusions are in agreement with the results of a study by Winkel et al. (29), in which the degree of relaxation of the amorphous state was probed by ambient pressure calorimetry. Their results indicate that high-density amorphous ice is in metastable equilibrium at 140 K and *P* ≤ ∼0.2 GPa and that an ultraviscous liquid state is assumed at 140 K and 0.07 GPa. Similarly, Handle et al. inferred that metastable equilibrium is almost reached at 0.2 GPa and 140 K and actually reached at 0.1 GPa and 140 K (16). In a further study Handle and Loerting concluded that the liquid state can be accessed below *T*_*x*_ at *P* < 0.4 GPa (17). The main difference between these studies and the present work is that we here locate the highest pressure at which HDL can be fully equilibrated without data extrapolation. From our data we conclude that this is the case at 0.3 GPa. The difference in terms of the maximum pressure, at which HDL can be observed, also reflects the different timescales inherent to the different methods in the different studies. At the low-pressure end, below 0.1 GPa equilibration of the high-density liquid is likely jeopardized by the formation of low-density amorphous nanodomains, as was shown by Tonauer et al. (28) and Handle et al. (30)

### Crystalline Remnants in the Amorphous Matrix.

**Fig. 4.**
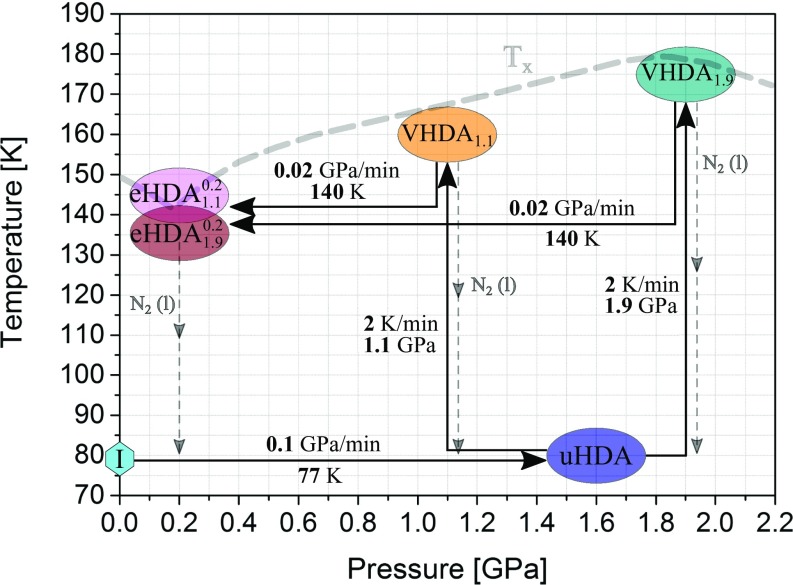
Schematic illustration of preparation routes for the different amorphous ices. For determination of *T*_*x*_, data from this work and from refs. 39–41 were considered.

To further examine the hypothesis of crystalline remnants influencing the crystallization behavior of amorphous ices we produced variants of eHDA. Instead of the method proposed originally by Winkel (31), namely decompression of VHDA_1.1_ at 140 K to 0.2 GPa (yielding $\text{eHD}{\text{A}}_{1.1}^{0.2}$), we decompressed VHDA_1.9_ at 140 K (yielding $\text{eHD}{\text{A}}_{1.9}^{0.2}$, see also Fig. 4). The idea of this preparation route is based on our earlier suggestion that VHDA_1.9_ contains fewer (nano)crystalline domains––if any at all––than VHDA_1.6_ (prepared at 1.6 GPa and 167 K) or VHDA_1.1_ (23). In fact, VHDA_1.1_ and VHDA_1.6_ exhibit a lower thermal stability against crystallization and a stronger tendency for parallel crystallization than VHDA_1.9_ (23). Thus, we proposed that in the case of VHDA_1.1_ the preparation conditions (i.e., time and/or temperature and/or pressure) are not sufficient for “crystallinity” to completely disappear and for the sample to fully amorphize. We further speculated that crystalline domains might also survive the decompression of VHDA_1.1_ at 140 K. Consequently, we surmised that $\text{eHD}{\text{A}}_{1.9}^{0.2}$ should then also exhibit fewer signs of crystallinity than $\text{eHD}{\text{A}}_{1.1}^{0.2}$ and *T*_*x*_$\left(\text{eHD}{\text{A}}_{1.9}^{0.2}\right)$ to be higher than *T*_*x*_$\left(\text{eHD}{\text{A}}_{1.1}^{0.2}\right)$. However, this is not the case: *T*_*x*_$\left(\text{eHD}{\text{A}}_{1.9}^{0.2}\right)$ is identical to *T*_*x*_$\left(\text{eHD}{\text{A}}_{1.1}^{0.2}\right)$ in the whole pressure range examined (Fig. 1). Thus, our results do not allow a conclusive assessment whether crystalline remnants survive the decompression at 140 K or not. Explanations for the unexpected observation could be that (*i*) no seeds are present in both types of eHDA, i.e., seeds disappear upon decompression, or (*ii*) new seeds are introduced in the decompression process that are identical for both types of eHDA. *T*_*x*_ of both eHDAs is constantly below *T*_*x*_(VHDA_1.9_) at *P* > 0.3 GPa. Moreover, the thermal stability of both eHDA types is also lower than that of VHDA_1.1_ (Fig. 1). This implies that the density of the amorphous matrix plays a key role in determining *T*_*x*_. The density difference between eHDA and VHDA persists up to *T*_*x*_ at *P* > 0.3 GPa, thereby ruling out the possibility of identical states being reached.

Regarding the latter finding of nonequilibrium upon heating before crystallization at *P* > 0.3 GPa we have performed further control experiments. By using 0.5 K min^−1^ rather than 2 K min^−1^ as heating rate we have provided a fourfold amount of time for the samples to equilibrate upon heating. However, as is shown in *SI Appendix*, Fig. S2 also this does not suffice to equilibrate the amorphous ices. The difference in *T*_*x*_ between eHDA and VHDA_1.9_ persists also for a heating rate of 0.5 K min^−1^. Furthermore, the difference remains small at 0.3 GPa, corroborating the idea that equilibration is possible at 0.3 GPa and below (*SI Appendix*, Fig. S2).

### Structural Examination.

**Fig. 5.**
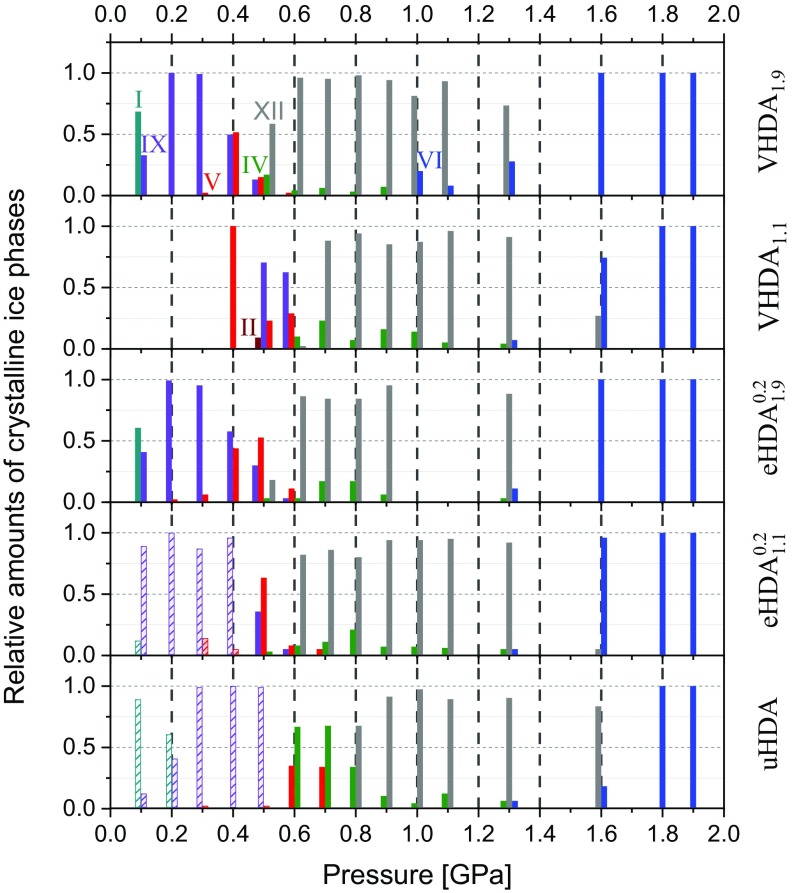
Schematic summary of all crystallization products from powder X-ray diffraction measurements, recorded at ∼80 K and ∼5 × 10^−1^ mbar. The bars indicate relative amounts of crystalline ices obtained from isobaric heating experiments at given pressures (abscissa) of the respective ice (right ordinate). The striped bars are from literature, refs. 8 and 20.

The crystallized samples are furthermore characterized structurally via powder X-ray diffraction (at ∼80 K and ∼5 × 10^−1^ mbar). The results are presented graphically in Fig. 5 (in tabular form see *SI Appendix*, Table S3). One can observe that depending on the pressure, the amorphous ices transform to a variety of different crystalline (singular) phases or phase mixtures (of up to four different ices) including ice I, ice II, ice IV, ice V, ice VI, ice IX, and ice XII. Salzmann et al. (24, 32, 33) were the first to label the transformation of an amorphous ice to more than one crystalline phase during a single crystallization event as “parallel reaction.” By just varying the heating rate they were able to change the relative amount of a given crystalline phase in a mixture from close to 0% to almost 100%. They consequently identified different crystallization processes associated with different rate constants taking place parallelly. Crystallization processes occurring with slower kinetics and at lower temperature were labeled as “type 1 kinetics” and the process occurring with faster kinetics and at higher temperature as “type 2 kinetics.” Here we observe similar tendencies: As visualized in Fig. 5 all amorphous ices experience parallel crystallization at almost all examined pressures at 2 K min^−1^. Notable exceptions occur at 1.8 and 1.9 GPa where the amorphous ices transform to stable ice VI exclusively.

eHDA and VHDA yield qualitatively similar crystallization products, especially eHDA_1.9_ and VHDA_1.9_ matching each other closely. Minor differences can be noticed for eHDA_1.1_ at 0.1 GPa and VHDA_1.1_ at 0.4 GPa. At 0.1 GPa mixtures of mainly ice I and ice IX as by-phase are formed. At pressures up to 0.6 GPa one observes varying mixtures of ices IX, V, II, IV, and XII. At 0.7–1.3 GPa a majority of ice XII crystallizes, with some ice IV at lower and some ice VI as by-phases at higher pressures. At 1.6 GPa predominantly ice VI forms in addition to minor amounts of ice XII. The only pressure range that allows for differentiation is 0.4–0.6 GPa. At 0.4 GPa VHDA_1.1_ transforms to pure ice V upon crystallization, while eHDA_1.1_ exhibits predominant formation of ice IX with ice V as by-phase. eHDA_1.9_ and VHDA_1.9_ on the other hand crystallize at 0.4 GPa to nearly equal amounts of ices V and IX (upper four panels in Fig. 5). At 0.5 GPa eHDA_1.9_ and VHDA_1.9_ form substantial amounts of ice XII, while for eHDA_1.1_ and VHDA_1.1_ ice XII is not formed at all.

uHDA by contrast yields qualitatively different crystallization products, deviating substantially from eHDA and VHDA. It crystallizes to predominantly ice I at 0.1 GPa (with some ice IX, lowest panel in Fig. 5) and similar amounts of ice I and ice IX at 0.2 GPa. At 0.5 GPa the main crystallization product is ice IX, again differing from the results of the other amorphous ices. Also at 0.6 and 0.7 GPa uHDA shows deviating behavior from the others, mainly ice IV forms instead of ice XII at these pressures. From 0.8 GPa onward the crystallized phase mixtures are rather similar for all. At 1.6 GPa, however, uHDA transforms to ice XII instead of ice VI. The results demonstrate that there is an additional channel in uHDA that lowers *T*_*x*_ compared with the other amorphous ices, especially below 0.8 GPa.

### Crystallization Times *t_cryst_*.

The above results naturally raise the question in which manner time frames of crystallization are connected to the crystallizing ice phases, or generally influenced by the experimental parameters. We estimate the crystallization times *t_cryst_* based on the difference between *t_cryst,onset_* and *t_cryst,end_* as shown in the insets in *SI Appendix*, Fig. S5. This evaluation is based on the assumption that the timescale for relaxation of the amorphous matrix and the timescale for crystallization are well separated. This assumption is valid both for *T*_*g*_ > *T*_*x*_ and *T*_*g*_ < *T*_*x*_, whereas for *T*_*g*_ ∼ *T*_*x*_ the two processes occur on similar timescales and cannot be separated in a straightforward manner. For $\text{eHD}{\text{A}}_{1.9}^{0.2}$ and VHDA_1.9_ the situation *T*_*g*_ < *T*_*x*_ is encountered at 0.1–0.3 GPa, as mentioned above, whereas the situation *T*_*g*_ > *T*_*x*_ is found for higher pressures. Only for 0.4 GPa the analysis is biased as both timescales are close to each other, so that the stepwise change at crystallization is overlapping with a simultaneous volume change based on relaxation. In other words, the volume changes slower as if crystallization alone was operative at ∼0.4 GPa. This can be noted by the slight increase in *t_cryst_* for $\text{eHD}{\text{A}}_{1.9}^{0.2}$ and VHDA_1.9_ at 0.4 GPa; see Fig. 3 and *SI Appendix*, Fig. S3, respectively.

Generally, crystallization times increase with increasing pressure. For $\text{eHD}{\text{A}}_{1.9}^{0.2}$ and VHDA_1.9_ they rise from close to 0 s to ∼200–300 s in the examined pressure range (Fig. 3). That is, even though the amorphous ices crystallize at higher temperatures at higher pressures the transformation process occurs more slowly. Crystallization times for uHDA in our study are comparable to eHDA and VHDA at *P* ≥ 0.8 GPa with the exception at 1.3 GPa, where VHDA_1.9_ crystallizes notably faster than $\text{eHD}{\text{A}}_{1.9}^{0.2}$ and uHDA. This is likely due to the pronounced formation of faster crystallizing ice VI (type 1 kinetics) in the case of VHDA_1.9_, whereas $\text{eHD}{\text{A}}_{1.9}^{0.2}$ and uHDA transform mainly to slower-crystallizing ice XII (type 2 kinetics) and very small amounts of ices IV and VI (see also Fig. 5). At *P* ≤ 0.7 GPa, however, uHDA “crystallizes” much slower than $\text{eHD}{\text{A}}_{1.9}^{0.2}$ and VHDA_1.9_. This holds true independent of whether similar or different polymorph mixtures crystallize. The main reason for the much broader crystallization steps for uHDA can be found in the processes contributing to it: For uHDA relaxation, type-1 and type-2 crystallization take place simultaneously at similar rates. Thus, the data points for uHDA do not represent crystallization alone, but a timescale for the combined processes.

One key aspect of Fig. 3 is the very short crystallization time of VHDA_1.9_ at *P* < 0.8 GPa. At 0.2 GPa *t_cryst_* amounts to about 2.5 s (*SI Appendix*, Fig. S5) at *T*_*x*_ = 149 K. At 1.9 GPa *T*_*x*_ is higher by about 30 K, but yet *t_cryst_* amounts to 190 s. This is indicative of a much higher self-diffusivity of H_2_O at low pressures, and we interpret this to be associated with much lower viscosity at *T*_*x*_. At *P* > 1 GPa the viscosity of amorphous ice at *T*_*x*_ is typical of solid-state viscosities on the order of 10^21^ Pa·s in this interpretation. Below 1 GPa the viscosity at *T*_*x*_ starts to drop by orders of magnitude, and at *P* ≤ 0.3 GPa the viscosity has dropped below 10^12^ Pa·s at *T*_*x*_ so that the HDL state is in fact reached.

Previous studies on the crystallization kinetics of amorphous solid water (H_2_O as well as D_2_O) were done on vapor-deposited amorphous ices at (sub)ambient pressure by monitoring the crystallization to cubic ice via infrared spectroscopy upon heating (34–36) or by electron diffraction (37). Crystallization rates for eHDA ($\text{eHD}{\text{A}}_{1.1}^{0.1}$ in our labeling) and VHDA (VHDA_1.1_ in our labeling) were reported only recently by Handle and Loerting (17), who obtained kinetic information from a fit procedure decomposing the measured volume changes into three contributions: relaxation and elastic expansion of the amorphous matrix and volume change caused by crystallization. Taking into account the sample geometry one can estimate rates of crystallization (in cm^3^/s or m/s, respectively) from Fig. 3, see *SI Appendix*, Fig. S3. The values obtained by Handle and Loerting (17) are in good agreement with ours for $\text{eHD}{\text{A}}_{1.9}^{0.2}$ and VHDA_1.9_, with an exception at 0.4 GPa (see figure 4 in ref. 17 and *SI Appendix*, Fig. S3). The crystallization rates of vapor-deposited amorphous ice at (sub)ambient pressure are much lower than the values for $\text{eHD}{\text{A}}_{1.9}^{0.2}$ and VHDA_1.9_ reported here. While growth rates at higher pressures *P* ≥ 1.6 GPa are only approximately two orders of magnitude larger near ∼180 K, the difference increases to seven to eight orders of magnitude at *P* ≤ 0.3 GPa and ∼140–150 K; see figure 3 in ref. 36 and *SI Appendix*, Fig. S3. That is, the formation of high-pressure crystalline ices is generally based on much faster kinetics than the formation of ice I at ambient pressure. The two orders of magnitude difference may be rationalized based on the shorter distances between atoms at high pressures, which implies shorter path lengths for the reaction coordinate leading to crystallization. The seven to eight orders of magnitude difference, however, requires a different molecular process, namely diffusion taking place in the liquid state under pressure. We reach the ultraviscous liquid HDL state at *P* ≤ 0.3 GPa, whereas the low-density liquid state has never been reached in UHV experiments of vapor-deposited thin films due to fast evaporation of the film near and above bulk water’s first *T*_*g*_ at 136 K.

## Summary and Conclusion

Our work strongly suggests that at *P* ≤ 0.3 GPa relaxed eHDA and VHDA reach the same equilibrated state before crystallization––namely HDL. This is indicated by the bifurcation point in *T*_*x*_ in Fig. 1 at 0.3 GPa. At *P* < ∼0.3 GPa the crystallization temperatures of eHDA and VHDA match each other closely, while at *P* > ∼0.3 GPa they differ. We infer this same equilibrated state to be of deeply supercooled liquid nature based on our observation of significant volumetric relaxation taking place near *T*_*g*_ (15, 17). That is, below 0.3 GPa crystallization does not impede the observation of the liquid (*T*_*g*_ < *T*_*x*_) while above 0.3 GPa timescales of crystallization become shorter than those of relaxation (*T*_*x*_ < *T*_*g*_). This interpretation is further substantiated by reported glass-to-liquid *T*_*g*_ values in the respective P/T region (14, 16). Our results also support the interpretation of an unrelaxed and structurally inhomogeneous nature for uHDA based on the considerably lower *T*_*x*_ values and the substantially different crystalline product phase composition at *P* < ∼0.9 GPa (Figs. 1 and 5).

Furthermore, our work defines a way to prepare amorphous ice that allows for water to stay in the noncrystalline state at higher temperatures than in previous work. At pressures *P* > ∼0.3 GPa VHDA_1.9_ is more stable against crystallization than all other amorphous ices. Δ*T*_*x*_ increases toward higher pressures, reaching ∼5 K at 1.8 GPa (Fig. 2 and *SI Appendix*, Table S2). The crystallization line *T*_*x*_(*P*) of VHDA_1.9_ can thus be regarded the true low-temperature boundary to water’s no man’s land. With respect to low-lying glass transition temperatures at 1.0 GPa (17–19) we conclude that they do not reflect a glass-to-liquid transition, but rather an orientational glass transition. That is, not the liberation of mainly translational degrees of freedom is involved, but rather the liberation of rotational degrees of freedom. If a glass-to-liquid transition was indeed the case at 1.0 GPa one would expect relaxation times to become so short with rising temperature that any distinction in the disordered states should be lost well before crystallization, and the *T*_*x*_ values should be the same in a margin of error. Our observation, however, is that *T*_*x*_ between eHDA and VHDA_1.9_ differs by about 6 K.

Finally, we present crystallization times of amorphous ices under high-pressure conditions and compare these data with recent work by Handle and Loerting (17) at elevated pressures and by Xu et al. (36) on amorphous solid water at UHV conditions. From the crystallization times *t_cryst_* the unrelaxed nature of uHDA can be inferred at *P* < ∼0.8 GPa (Fig. 3). While in the case of eHDA and VHDA crystallization takes place on the order of seconds, it takes much longer for uHDA. The very high crystallization rates of eHDA and VHDA at the low-pressure end (see *SI Appendix*, Fig. S3), and the increase toward higher pressure supports the idea that the mechanism of crystallization changes. Rather than the solid–solid transformation that is incurred at high pressures, the access of less viscous states accelerates the crystallization at low pressures.

## Materials and Methods

Fig. 4 demonstrates the five routes of preparation by which amorphous ices were obtained and introduces the nomenclature used in this work. uHDA was prepared by PIA as originally described by Mishima et al. (1). Two variants of VHDA were prepared for this work, one according to the original work of Loerting et al. (labeled here VHDA_1.1_) (38), the other one by heating to 175 K at 1.9 GPa (labeled here VHDA_1.9_). The two variants of eHDA were prepared by decompressing VHDA_1.1_ or VHDA_1.9_ at 140 K to an end pressure of 0.2 GPa (labeled here $\text{eHD}{\text{A}}_{1.1}^{0.2}$ and $\text{eHD}{\text{A}}_{1.9}^{0.2}$, respectively). Sample preparation was done in the same way as described in our earlier work (22, 23). In short, 500 μL of liquid water (ultrapure) are pipetted into a precooled indium cylinder upon which polycrystalline hexagonal ice I_h_ forms. The sample is compressed/decompressed using a commercial material testing machine (ZWICK model BZ100/TL3S) in a controlled-temperature environment. The material testing machine is operated as a pressure device as well as a dilatometer, making it possible to record the change in sample volume with time/temperature in situ.* Friction effects were determined to be negligible in our setup: Nominal pressures calculated from the ratio of load and sample cross-section were found to be identical with actual pressures as determined from known phase transitions between stable ice phases. Similarly, temperatures were calibrated against these phase transitions and found to deviate by no more than 0.2 K. Reproducibility of temperatures is better than ±0.1 K. Structural characterization of the quench-recovered crystallized products is performed via powder X-ray diffraction at ∼80 K in vacuo (Siemens, model D5000 and Bruker, model D8 Advance; in both cases Cu *Kα* radiation is employed, λ = 1.5406 Å). Diffractograms are recorded in θ-θ geometry.

Onset crystallization temperatures *T*_*x*_ and crystalline phase compositions were determined as described in our previous studies on the topic (22, 23). A detailed description can be found in *SI Appendix*. In principle, onset temperatures of crystallization were identified by an abrupt change in the volume-versus-temperature plot (*SI Appendix*, Fig. S4). This step-like volume change is indicative of the transformation from an amorphous ice of a given density *ρ_amor._*(*T,P*) to a crystalline phase/phase mixture of a different density *ρ_cryst_* (*T,P*). The standard deviation determined from several sets of identical isobaric heating experiments at different pressures is ±0.2 K (23).

## Footnotes

*Seidl M, et al., Sixth Zwick Academia Day, June 2, 2015, Zurich, Switzerland. Available at http://www.loerting.at/publications/seidl15-zwick.pdf.
